# A New Slow Releasing, H_2_S Generating Compound, GYY4137 Relaxes Spontaneous and Oxytocin-Stimulated Contractions of Human and Rat Pregnant Myometrium

**DOI:** 10.1371/journal.pone.0046278

**Published:** 2012-09-27

**Authors:** Hayley Robinson, Susan Wray

**Affiliations:** Department of Cellular and Molecular Physiology, Institute of Translational Medicine, University of Liverpool, Liverpool, United Kingdom; Fudan University, China

## Abstract

Better tocolytics are required to help prevent preterm labour. The gaseotransmitter Hydrogen sulphide (H_2_S) has been shown to reduce myometrial contractility and thus is of potential interest. However previous studies used NaHS, which is toxic and releases H_2_S as a non-physiological bolus and thus alternative H_2_S donors are sought. GYY4137 has been developed to slowly release H_2_S and hence better reflect endogenous physiological release. We have examined its effects on spontaneous and oxytocin-stimulated contractility and compared them to NaHS, in human and rat myometrium, throughout gestation. The effects on contractility in response to GYY4137 (1 nM–1 mM) and NaHS (1 mM) were examined on myometrial strips from, biopsies of women undergoing elective caesarean section or hysterectomy, and from non-pregnant, 14, 18, 22 day (term) gestation or labouring rats. In pregnant rat and human myometrium dose-dependent and significant decreases in spontaneous contractions were seen with increasing concentrations of GYY4137, which also reduced underlying Ca transients. GYY4137 and NaHS significantly reduced oxytocin-stimulated and high-K depolarised contractions as well as spontaneous activity. Their inhibitory effects increased as gestation advanced, but were abruptly reversed in labour. Glibenclamide, an inhibitor of ATP-sensitive potassium (K_ATP_) channels, abolished the inhibitory effect of GYY4137. These data suggest (i) H_2_S contributes to uterine quiescence from mid-gestation until labor, (ii) that H_2_S affects L-type calcium channels and K_ATP_ channels reducing Ca entry and thereby myometrial contractions, (iii) add to the evidence that H_2_S plays a physiological role in relaxing myometrium, and thus (iv) H_2_S is an attractive target for therapeutic manipulation of human myometrial contractility.

## Introduction

Hydrogen sulphide (H_2_S) is a gaseous signalling molecule that has been implicated in several physiological and pathophysiological processes from long term potentiation [1] and inflammation [2], to smooth muscle contractility [3], [4], [5], [6]. Two cytosolic enzymes, cystathionine β-synthase (CBS) and cystathionine γ-lyase (CSE) [7] act on the sulphur containing amino acids, cysteine and homo-cysteine, to produce H_2_S [8], [9]. At least two enzymes have been identified that degrade H_2_S to thiosulfate and sulphate, thiosulfate sulphur transferase and Thiol S- methyltransferase [3], [8], [10]. Thus H_2_S will be physiologically regulated within cells and rapid rates of turnover enable it to function as a signalling molecule [11].

The effects of H_2_S have been examined in several smooth muscles and decreased contraction is the most common finding e.g. vas deferens, [3], blood vessels [12] GI tract [3], [4], [5]. The mechanism by which H_2_S produces its effects on smooth muscle contractility are not clear, although K_ATP_ channels have been implicated in some studies [13], [14], [15], [16]. Other studies however have found no role for K_ATP_ channels [5], [17], [18]. In the myometrium although K_ATP_ channels are expressed [19] they so far appear to have only a limited functional importance compared to voltage dependent K channels [20], [21], [22], thus other targets for H_2_S may be important in the myometrium. Changes in intracellular [Ca] are known to underlie contractility changes in response to agonists and tocolytics in myometrium [22], [23], [24]. Recently, a study in cardiomyocytes suggested, H_2_S inhibits L-type [Ca] channels through sulfhydration, as NaHS decreased the functional free sulfhydryl groups in the channels [25]. In non-contracting (butanedione monoxime treated) cerebral artery, Tian et al, [26] used fluo-4 and showed decreases in Ca levels as NaHS was increased from 0.1 to 1 mM, and suggested that NaHS relaxes these vessels by reducing L-type Ca current. There have however been no simultaneous measurements of the changes of intracellular Ca that occur when changes in contraction result with H_2_S production in any tissue, and hence its role in the mechanisms of H_2_S’s effects is unclear. Understanding how H_2_S affects Ca signalling in smooth muscle will provide further insight into how H_2_S can affect force.

There is a pressing need to better understand how uterine contractility is controlled and to develop better tocolytics to reduce the morbidity and mortality associated with pre-term delivery [27], [28]. Thus an endogenous molecule that can reduce contractility is of interest. It has already been shown that the uterus possesses the enzymes to produce H_2_S from L-cysteine, and reports have shown H_2_S to be able to reduce contractions of myometrium from rat and human [6], [29], [30]. Thus alterations of H_2_S levels may be an attractive target for therapeutic manipulation in problematic labours. It is not clear however if the effects of H_2_S are gestationally dependent, which would indicate that H_2_S is part of the mechanism maintaining uterine quiescence and governing the switch to labour onset, or if it remains at an unchanged constitutive level in myometrium.

The previous studies investigating H_2_S in myometrium used addition of NaHS as a means of producing H_2_S. This will produce H_2_S in a large, rapid bolus and thus it may be questioned how well this simulates the physiological condition. In addition because of its potential lethality, it is unlikely that NaHS will be a useful therapeutic tool. Recently a novel H_2_S generating compound, GYY4137 (morpholin-4-ium 4 methoxphenyl (morpholino) phosphinodithionate) has been developed. It slowly releases H_2_S, both *in vitro* and *in vivo* [31], and has been shown to slowly relax aortic rings and *in vivo* to cause vasodilation and act as an anti-hypertensive [31]. To the best of our knowledge this more physiological approach to the study of H_2_S in myometrium has not been examined. This in turn limits information on which to judge the clinical potential usefulness of H_2_S manipulation in controlling uterine activity. In order to increase mechanistic understanding of how H_2_S reduces uterine contractility, we have also made simultaneous measurements of changes in intracellular Ca and force [32].

The aims of our study were therefore to determine: (1) the effects of GYY4137 on contractions of human and rat myometrium, (2) how responses of the myometrium to H_2_S vary with gestational state, (3) the effects of glibenclamide on GYY4137-induced changes in contractility; (4) the effects of H_2_S produced via NaHS and GYY4137 on spontaneous, oxytocin and high K depolarization stimulated contractions, and (5) the effect of GYY4137 on myometrial Ca signals.

## Methods

### Ethics Statement

This study was given a favourable ethical opinion and approved by the North West Liverpool Research Ethics Committee (REC refs 10/H1002/49, 09/H1005/55 and 11/H1005/4) and by the Research and Development Director of Liverpool Women’s NHS Foundation Trust, Liverpool, UK. All women provided written informed consent for the collection of samples and subsequent analysis. The animals used for these studies were maintained and cared for under the University of Liverpool Animal care and Use Committee. Animals were humanely killed and tissue removed in accordance with UK legislation. All protocols were approved by the Liverpool University Animal Use and Care Committee.

### Tissues

Strips of longitudinal myometrium free of circular muscle (∼ 1×5 mm) were dissected from the uterus of humanely killed non-pregnant, 14 day, 18 day and 22 day gestation and labouring Wistar rats [28]. The gestation of the rat was defined from day 0, when the male was placed in the cage to mate. Human myometrial strips were dissected from biopsies obtained with informed consent and ethical approval from women undergoing an elective term caesarean section (means gestational age 39 weeks, mean maternal age, 31; range 22–41 years, N = 15) or pre-menopausal hysterectomy (mean age, 40; range 27–48 years, N = 12). Indications for caesarean section included maternal request, previous traumatic vaginal delivery, previous caesarean section or breech presentation. None of the women included in this study had underlying diseases (hypertension, diabetes, pre-eclampsia, intrauterine growth restriction etc.). Indications for hysterectomy were menorrhagia, fibroids or prolapse. Biopsies were obtained from the upper lip of the lower segment uterine incision at caesarean section [33] and from corresponding macroscopic normal area of the uterus at hysterectomy.

### Solutions

All chemicals were obtained through Sigma (UK), apart from GYY4137, which was obtained from Santa Cruz biotechnology, USA, NaHS, obtained from Alfa Aesar, UK and Indo-1, Invitrogen, UK. The composition of Physiological Saline Solution (PSS) was as follows (mM): 154 NaCl, 5.1 KCl, 0.12 MgSO_4_7H_2_O, 10.9 HEPES, 8 Glucose, 2 CaCl_2_, pH 7.4. In some experiments to depolarize the tissue, the KCl in the PSS was increased to 40 mM and NaCl reduced equivalently. In some experiments, 0.5 nM oxytocin was added to the PSS to study oxytocin induced contractions. The H_2_S forming solutions were made in PSS at 1 mM for NaHS and 1 n M, 1 µM, 0.1 and 1 mM for GYY4137, re- pH ’d to 7.4. [34]. Both GYY4137 and NaHS were made and incubations performed in a fume cupboard at 37°C. Glibenclamide was used at 10 µM.

### Ca^2+^ and Force Measurements

The longitudinal strips of myometrium were clipped using aluminium hooks. One clip was attached to a fixed hook in a small tissue bath, which was situated above the objective of an inverted microscope, and the other clip to a force transducer [35]. Strips at a resting tension of 2 mN were then superfused with the PSS at 37°C, pH 7.4 at 2 ml/min. For simultaneous measurement of calcium the strips were loaded with 50 µg Indo-1 AM and 50 µl pluronic Acid and DMSO mix dissolved in 4 ml PSS for 3–4 hours on a spinning carousel at 21°C [36]. These tissues were rinsed and then transferred to the tissue bath and transducer as above, and the indo-1 was excited at 350 nm. The emitted light at 400 nm and 500 nm was recorded on PMTs at 100 Hz. The ratio of 400∶500 nm indo-1 fluorescence gives the changes in intracellular Ca^2+^ [37]. Incubation with Indo-1 and DMSO (also used to dissolve glibenclamide) has been shown not to affect contractility [38].

### Protocol

Contractile activity was seen in all myometrial strips within 60 minutes for rat and 3 hours for human after perfusion with PSS [39]. The strips were allowed to contract spontaneously and an equilibrium period of at least 30 min with stable contractions was obtained before incubation in any chemical. After recording control activity, the effect of 45 minute exposure to NaHS, GYY4137 or control (PSS) solution on uterine activity was examined by placing the strip in an eppendorff with the agent, at 37°C within a fume hood due to the toxicity of H_2_S, if not contained. The tissues were then carefully re-attached to the tension transducer, superfused with PSS and contractility again recorded. The same was performed for glibenclamide experiments only the control activity was exposed to 10 µM glibenclamide as well as during incubation in GYY4137 1 mM or PSS. Each concentration of drug was obtained on a separate strip of myometrium.

### Data Analysis

Contractions were analysed for amplitude, frequency, and area under the curve, (AUC, in arbitrary units, au) for; 10 minutes, rat data; 30 min, human data (to accommodate the slower rate of contractions), and; 15 minutes, high K, before and after H_2_S forming solution incubation, using origin 8 [40]. Each strip tested for the effect of each H_2_S producer, had a paired control response in PSS rather than test solution. After incubation the contractions were assessed 5 minutes after re-attachment. Student’s t tests were performed to compare two groups. Anova with Bonferroni post hoc tests were used to compare more than two groups. P was taken as showing a significant difference when P<0.05. Each experiment was performed on a separate strip from a different biopsies or rat.

## Results

### Control Protocol and the Effect of NaHS in Term Pregnant Rat Myometrium

**Figure 1:**
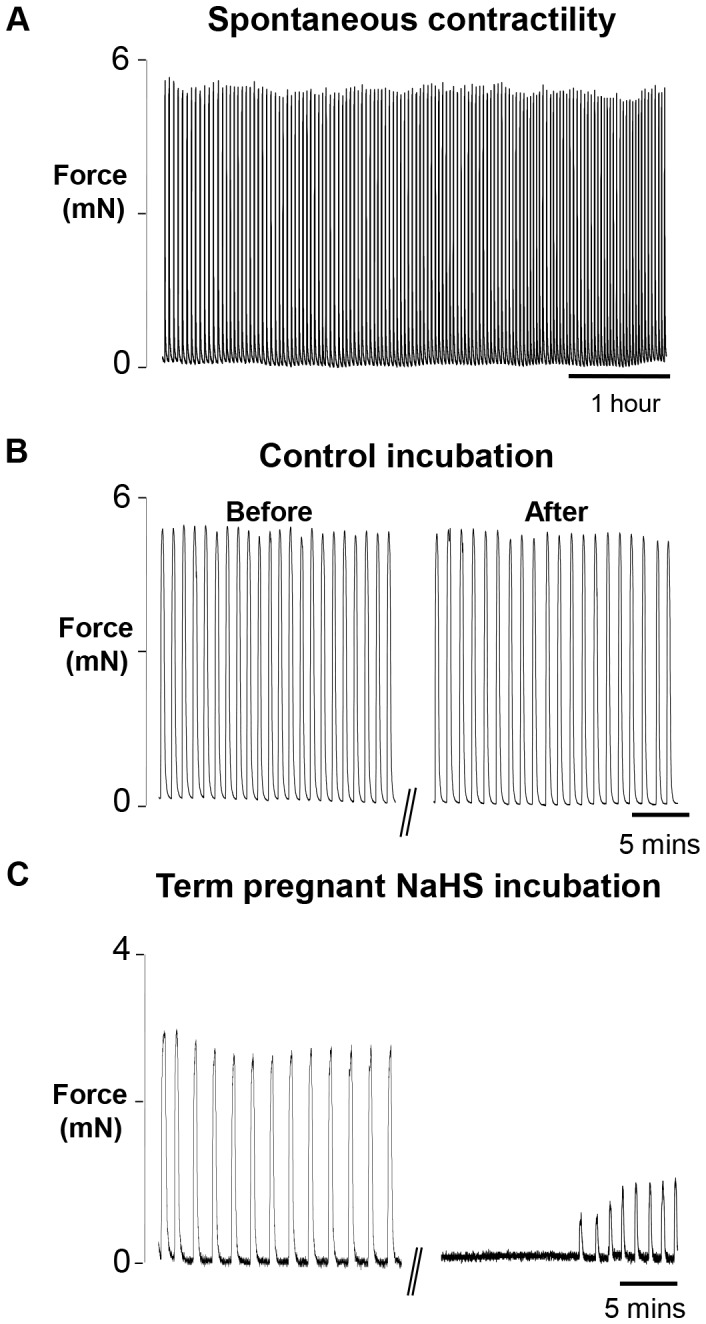
Effect of NaHS on myometrial contractility. Representative isometric recordings of spontaneously contracting myometrial strips obtained from 22 day gestation rat (term). (A) Continuous spontaneously active strip. (B) Control contractility before and after incubation in physiological saline solution (PSS) and re-attachment of strip. (C) Contractility before and after incubation in 1 mM NaHS and re-attachment of strip. Strips in this and subsequent figures were placed under a resting tension of 2 mN and superfused continually with physiological saline solution, pH 7.4 at 37°C before and after the 45 minute incubation periods, which were also at pH 7.4 at 37°C.

Due to the great toxicity of H_2_S the tissue strips with the H_2_S producing solutions were incubated in a fume cupboard and then re-attached via their clips, to the tension transducer at the end of the incubation period. It was therefore necessary to show that under control conditions i.e. incubation with PSS and re-attaching, no significant changes in contractile parameters were found when contractions were re-established. Figure 1 shows that this was the case. As can be seen in Figure 1A, steady rhythmic spontaneous contractions could be recorded under control conditions from 22 day (term) rat myometrium for many hours. Figure 1B, shows contractions before and after a 45 minute control incubation and re-attachment, typical of 6 other control strips. Analysis of the 7 strips showed that there were no significant changes to any of the parameters of contractions (Table 1). Figure 1C shows a strip which had been incubated in 1 mM NaHS and clear effects on contraction are apparent. The mean data for contraction amplitude, frequency and AUC measured over 10 minutes, after incubation with NaHS, compared with the immediate control period are shown in Table 1; significant reductions in all three parameters of contraction occurred. Figure 1C also shows that contractions return after a brief time upon re-attachment and superfusion in control solution.

**Table 1:** Changes in contractile parameters in response to NaHS, GYY4137 and GYY4137 with Glibenclamide, in term pregnant rat myometrium.

Parameter measured	Control (% ± SE, n = 7)	NaHS incubated(%± SE, n = 7)	GYY4137 incubated(% ± SE, n = 6)	GYY4137+ Glibenclamide(%± SE, n = 4)
**Contraction Amplitude**	99±2%	15±8%*	34±6%*	87±13%
**Frequency**	100±7%	23±9%*	42±10%*	101±27%
**AUC**	95±5%	16±13%*	22±10%*	98±21%

Term (day 22) rat myometrial strips were studied from 4–7 animals. After baseline values were obtained (10 minute period immediately before incubation in experimental solutions, 100%), tissues were incubated in either physiological saline (control) or the solutions indicated, for 45 minutes and then the parameters of contraction re-measured, and expressed as the percent of baseline values (i.e. paired data).Values are means ± s.e.m.

* represents significant differences in contractility compared to preceding control period (p<0.05, t-test). AUC; area under the curve.

### Dose Dependent Effects of GYY4137 on Spontaneous Contractions in Term Pregnant Rat Myometrium

**Figure 2:**
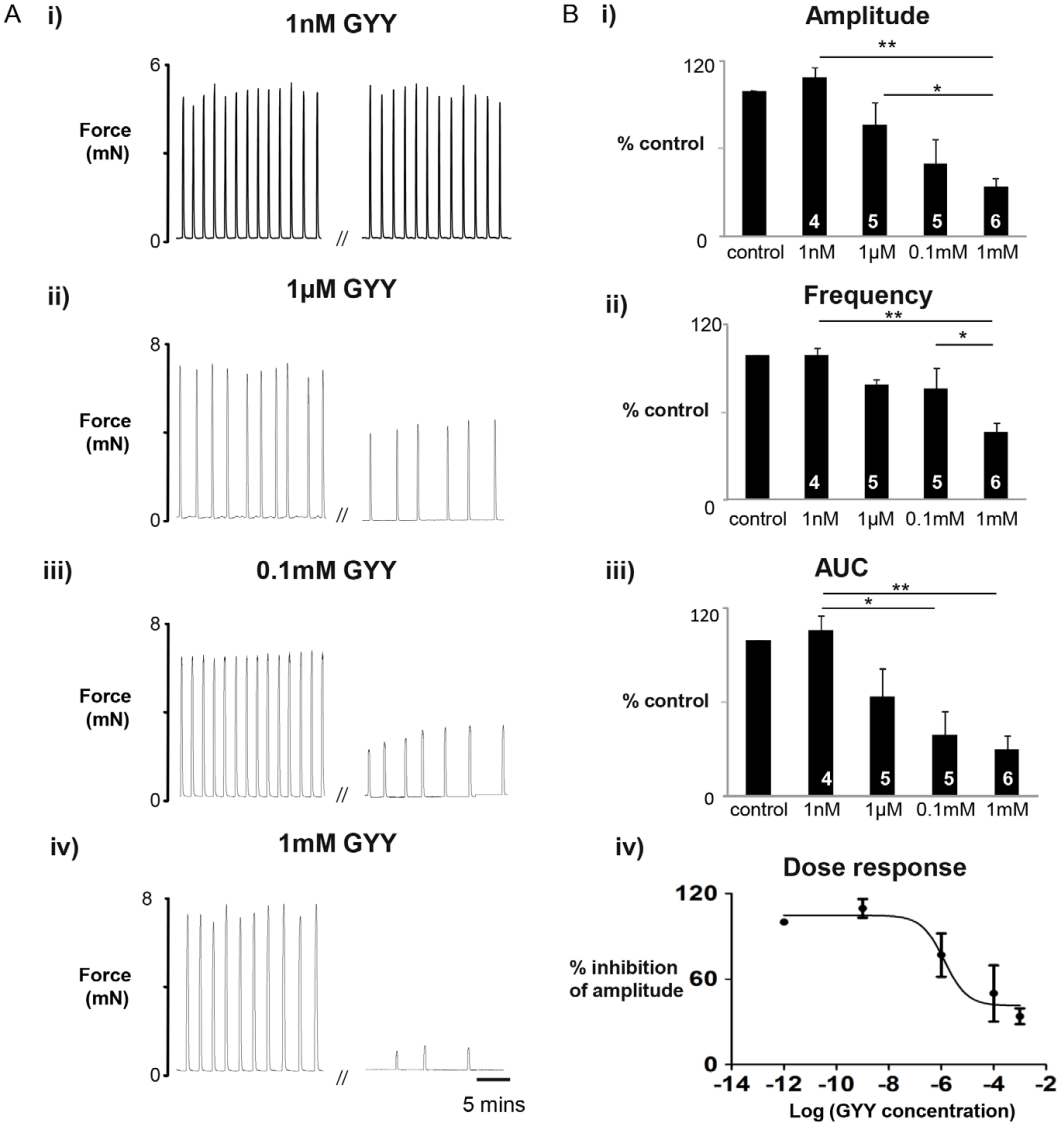
Dose dependency of GYY4137 in term pregnant rat. (**A**) Representative isometric recordings of spontaneously contracting myometrial strips obtained from 22 day gestation rat, before and after 45 minute incubations in i) 1 nM, ii)1 µM, iii) 0.1 mM, iv) 1 mM GYY4137 (GYY). (**B**) Mean data ± s.e.m, denoted by error bars, showing the dose dependent decrease in i) Amplitude, ii) Frequency, iii) AUC in response to GYY. Iv) the dose response curve for the % inhibition of amplitude. Values within bars indicate n-numbers. * represents P<0.05, ** represents p<0.01, using Anova with Bonferroni *post hoc* tests.

Having established a robust protocol we proceeded to determine the effects of GYY4137 on myometrial contractility. Concentrations of GYY4137 from 1 nM to 1 mM were examined in term (day 22) pregnant rat myometrium. As seen in the original traces, (Figure 2A), GYY4137 dose dependently inhibited the spontaneous phasic contractile activity of the myometrium. Figure 2Bi–iii shows the mean data for the parameters of contraction and in Figure 2Biv, the fitted curve to amplitude, giving an EC_50_ of 1.3±0.2 µM for GYY4137.

### Gestational Dependent Effects of GYY4137 and NaHS

**Figure 3:**
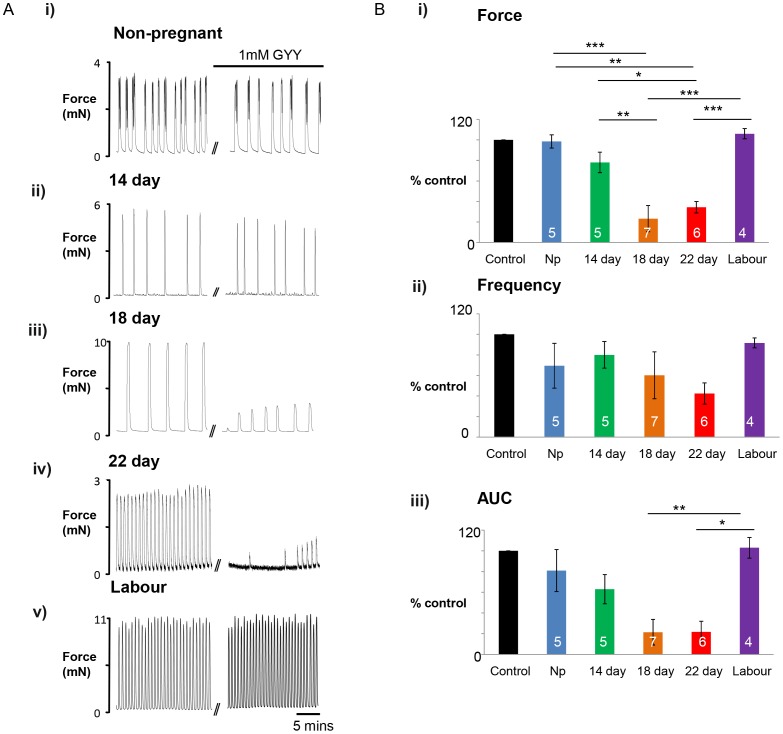
Rat myometrial contractility changes over gestation in response to GYY4137. (**A**) Representative isometric recordings of spontaneously contracting myometrial strips obtained from i) non-pregnant (NP), ii) 14 day, iii) 18 day, iv)22 day gestation and v) in labour rats before and after incubation in 1 mM GYY4137 (GYY). (**B**) Mean data ± s.e.m, denoted by error bars, of the gestational dependent decrease in i) Amplitude, ii) Frequency, iii) AUC in response to GYY. Values represent Means ± s.e.m, denoted by error bars. Values within bars indicate n-numbers. * represents P<0.05, ** represents p<0.01, *** represents p<0.01, using Anova with Bonferroni *post hoc* tests.

**Figure 4:**
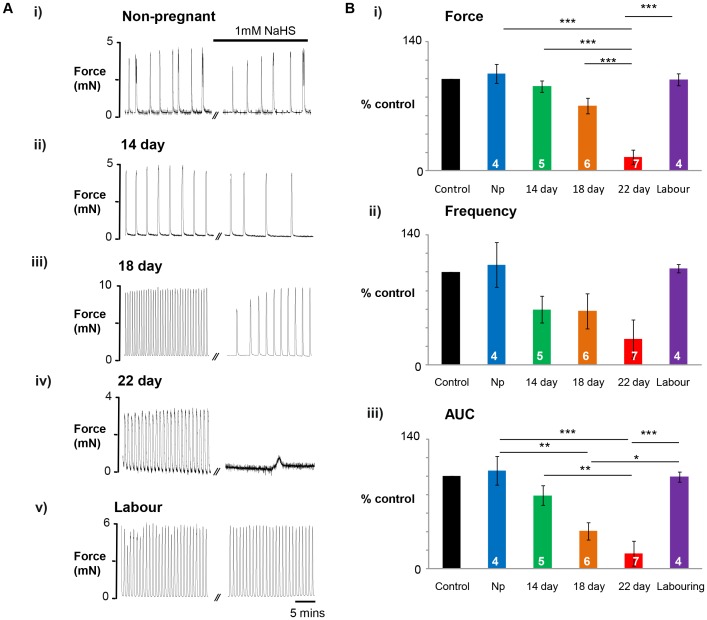
Rat myometrial contractility changes over gestation in response to NaHS. (**A**) Representative isometric recordings of spontaneously contracting myometrial strips obtained from i) non-pregnant (NP), ii) 14 day, iii) 18 day, iv) 22 day gestation and v) in labour rats. (**B**) Mean data ± s.e.m, denoted by error bars, of the gestational dependent decrease in i) Amplitude, ii) Frequency, iii) AUC in response to NaHS. Values within bars indicate n-numbers. * represents P<0.05, ** represents p<0.01, *** represents p<0.01, using Anova with Bonferroni *post hoc* tests.

Having shown that GYY4137 can reduce contractions of term pregnant myometrium we next examined if its efficacy varied throughout pregnancy, and as no data was available on this point for NaHS, we also investigated if its effects varied with gestational state. Typical examples of the effects of 1–mM GYY4137 (n 4–7) and NaHS (n 4–7) from non-pregnant, 14, 18 and 22 day pregnant and labouring rat tissue are shown in Figures 3A and 4A respectively.

It can be seen that GYY4137 has little effect on non-pregnant myometrium, (Figures 3Ai). Increasing effects of GYY4137 on contractility as term progressed were found (Figure 3Aii–iv). There was a marked reversal of the inhibitory effect of GYY4137 once labour was initiated, i.e. No effect on spontaneous contractions was found (Figure 3Av). As shown in the mean data, (Figure 3B), amplitude, frequency of contractions and AUC are progressively reduced by GYY4137 from mid-gestation up until labour onset. The effects were small at day 14 and increased as gestation advanced. Compared to non-pregnant and labouring tissue, which showed no significant changes with GYY4137 incubation, the effects of GYY4137 were significant at day 18 and 22 of gestation.

As we show in Figure 4A the effects of NaHS also increased as gestation advanced. As with GYY4137 there was no significant effect on the non-pregnant (Figure 4i) or labouring (Figure 4v) myometrium. The mean data for the effects of NaHS throughout gestation are shown in Figure 4B and the significant effects compared to non-pregnant myometrium can be seen at 18 and 22 days gestation.

### Effects on Calcium Entry and Calcium Signalling

As the above data show very clear reductions in the strength of myometrial contractions in the presence of H_2_S we next determined if Ca channels and transients are affected by H_2_S producers in two ways. Firstly, we used high K to depolarize the myometrium and open voltage gated Ca channels [39]. If the response to depolarization is unaltered by the H_2_S donors then this would indicate that their effects were on the normal processes leading to membrane depolarization. Secondly we have made direct measurements of intracellular Ca simultaneously with force in the absence and presence of GYY4137. If Ca entry is decreased, then this should be apparent in the associated Ca transients [40].

**Figure 5:**
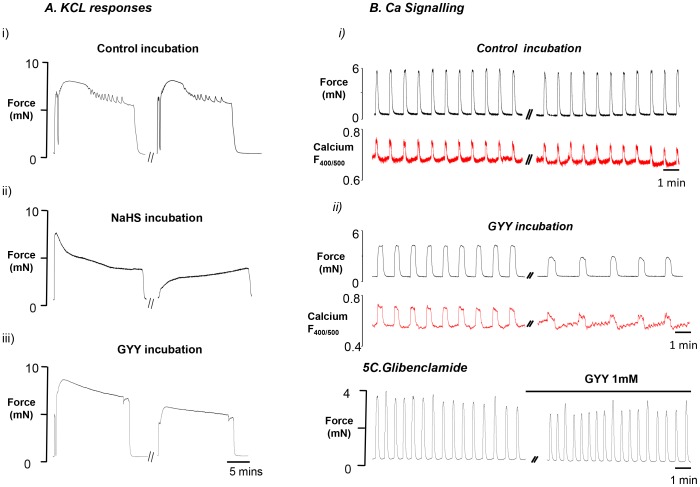
Effects on calcium entry and calcium signalling. (**A**) High K (40 mM) depolarisation, 15 minutes, of term pregnant rat myometrial strips and the effects in the presence of i) physiological saline solution (PSS), ii) NaHS (n = 6) iii) GYY4137 (GYY, n = 8). (**B**) Recording of force and intracellular Ca (from Indo-1–emitted fluorescence F400∶500), measured simultaneously in spontaneously contracting myometrial strips dissected from term rat myometrium before and after incubation in i) Control (PSS), ii) GYY 1 mM (n = 5). (**C**) K_ATP_ channel inhibitor glibenclamide (10 µM) was applied to rat myometrial strips 20 minutes before and during the 45 minute GYY (1 mM, n = 4) incubation period. All solutions were used at 37°C and pH 7.4.

Depolarisation using KCl for 15 minute was examined before and after 45 minute incubation in NaHS (n = 6) and GYY4137 (n = 8). In day 22 pregnant rats, the two successive applications of high K^+^, produced very similar contractile responses; a rapid rise in force which plateaus and shows only a small decrement until the tissue is returned to control solution (Figure 5Ai). As shown in Figures 5A ii and iii, both H_2_S producers inhibited the amplitude of the KCl contractions significantly (43±10%, NaHS 82±6% GYY4137) and AUC (34±4%, NaHS 84±7% GYY4137) compared to control (95±5%, 105±3% respectively).

The effects of 1 mM GYY4137 on Ca signalling on day 22 of gestation were examined. As Figure 5B clearly shows spontaneous Ca transients (indo-1 fluorescence) underlie the phasic contractions of the myometrium. As before, GYY4137 produced significant decreases in contraction amplitude, which as can be seen in Figure 5Bii, are accompanied by a decrease in the amplitude of Ca transients to 76±8%, (*n* = 5).

### Effects of K_ATP_ Channel Inhibition on GYY4137-induced Changes in Contractility

Previous studies have demonstrated that K_ATP_ channels are involved in H_2_S modulation of smooth muscle tone [15], [16] but this has not been studied in rat uterus. In 4 paired experiments the effects of GYY4137 in the presence of glibenclamide, (10 µM) a blocker of K_ATP_ channels was investigated. As previously found [20] glibenclamide had little effect on spontaneous contractions (not shown). Incubation of GYY4137 in the continued presence of glibenclamide had little effect on the parameters of contraction within rat myometrium (Figure 5C). As can be seen in Table 1, GYY4137 did not produce any significant effects when glibenclamide was present.

### Effects of GYY4137 and NaHS on Spontaneous and Oxytocin-stimulated Contractions of Human Myometrium

**Figure 6:**
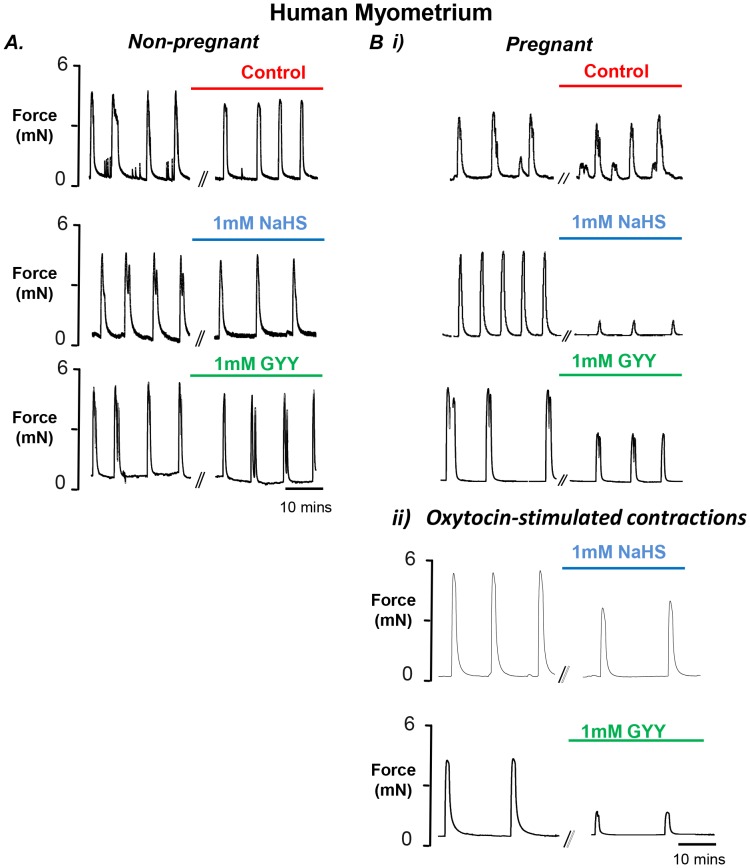
Comparison of non-pregnant and term pregnant human myometrial contractility in response to H_2_S producers NaHS and GYY4137. Representative isometric recordings of spontaneously contracting myometrial strips obtained from (**A**) non-pregnant, and (**B**) term pregnant non-labouring human tissue, both i) spontaneous ii) oxytocin-stimulated (0.5 nM) contractions were assessed. Strips were placed under a resting tension of 2 mN and superfused continually with physiological saline solution (pH 7.4) at 37°C before and after 45 minute incubations in physiological saline solution (control, represented in red), 1 mM NaHS (blue), 1 mM GYY4137 (GYY) (green) all at a pH 7.4 and at 37°C.

Having established that GYY4137 and NaHS could significantly reduce force in term but not non-pregnant rat myometrium, we next investigated their effects on human myometrium. As shown in Figure 6A neither NaHS (n = 6) nor GYY4137 (n = 5), had any significant effects on non-pregnant human tissues. In contrast, as shown in Figure 6Bi, both produced clear effects on term human myometrium and significant reductions in force (Table 2). Thus both H_2_S producers can significantly reduce force in term-pregnant human myometrium.

**Table 2:** Changes in pregnant human myometrial contractile parameters in response to NaHS, and GYY4137 incubations with and without oxytocin.

Parameter measured	Control (%)	NaHS incubated(%± SE, n = 7)	NaHS +Oxytocin(%± SE, n = 7)	GYY4137 incubated(%± SE, n = 6)	GYY4137+Oxytocin(%± SE, n = 5)
**Contraction Amplitude**	100	10±4%* ‡	40±9%*	35±14%*	33±14%
**Frequency**	100	23±9%* ‡	50±10%*	48±19%*	76±26%
**AUC**	100	3±2%* ‡	22±7%*	23±10%*	29±12%*

After baseline values were obtained (30 minute period immediately before incubation in experimental solutions,100%), tissues were incubated in either physiological saline (control) or the solutions indicated, for 45 minutes and then the parameters of contraction re-measured, and expressed as the percent of baseline values (i.e. paired data).Values are means ± s.e.m.

* represents significant differences in contractility compared to preceding control period.(p<0.05, t-test).

‡ represents significant reduction in spontaneous contractility when compared to, in the presence of oxytocin (0.5 nM) along with either GYY4137 or NaHS. AUC; area under the curve.

*In vivo* term myometrium will be stimulated by circulating oxytocin thus it could be posited that this stimulation prevents the effects of H_2_S. We therefore investigated if GYY4137 and NaHS could reduce contractility in term myometrium stimulated by oxytocin. Oxytocin produced a clear increase in the force and frequency of contractions (not shown) upon which the effects of, NaHS, (n = 7) and GYY4137 (n = 5) were tested (Figure 6Bii). Both compounds reduced significantly the parameters of contraction in all samples. When compared to spontaneous activity however it can be seen (Table 2) that their effects were not as large.

## Discussion

The present study is the first to investigate the effects of the novel H_2_S generator GYY4137 in a non-vascular smooth muscle, the myometrium. We studied its effects in rat and human myometrium and report here that: i) GYY4137 causes a concentration-dependent reduction in contractility of myometrium, ii) The ability of H_2_S to inhibit contraction is not constant but rather is greatest close to term before disappearing during labour iii) GYY4137 and NaHS significantly reduced contractility in pregnant but not non-pregnant human myometrium, iv) H_2_S significantly reduced tonic force produced by high-K depolarization and oxytocin-stimulated contractions, and v) GYY4137 reduced the intracellular Ca transients underlying contractions and inhibition of K_ATP_ channels prevented the effects of GYY4137. Together these data suggest H_2_S affects both membrane potential and L-type Ca channels to relax myometrium and that physiologically, H_2_S levels may be altered during gestation to contribute towards myometrial quiescence until labour. This suggestion is supported by recent findings reporting that H_2_S production is decreased within human term labouring myometrium compared to non labouring myometrium [30].

### Experimental Conditions and Protocols

GYY4137, like NaHS, releases H_2_S when in aqueous solutions such as PSS, but was developed to release it with a slower and more prolonged time course than that obtained with sulphide salts [31], [41]. Measurements of H_2_S *in vivo* and *in vitro* confirmed a release of H_2_S with GYY4137 taking several minutes to peak, whereas NaHS produces a larger, more or less instantaneous release of H*_2_*S. In subsequent work it was confirmed that H_2_S release from GYY4137 was 10% of that observed with NaHS, but was sustained, [42] and that a structural analogue, ZYJ1122, which lacked sulphur, was without effect. Our incubations were performed in a fume cupboard and tissue then transferred to the experimental rig for force and other measurements. We also waited five minutes after re-attachment of the tissue to the force transducer, to allow the tissue to settle and wash off of the H_2_S producing compounds. This is likely therefore to have resulted in an under-estimation of the effects of H_2_S, and suggests *in vivo* that GYY4137 will be more potent than measured in our *in vitro* studies. As seen in many of the figures, force builds up throughout the period after incubation, presumably as the H_2_S is volatilized and oxidized. [34]. Notwithstanding these experimental conditions clear effects of GYY4137 are apparent. Consistent with previous work [6], [29] we found that NaHS decreased or even abolished spontaneous contractility in rat and human myometrium.

### GYY4137 and Smooth Muscle

In the only other study on smooth muscle function, Li et al showed GYY4137 could relax contraction of aortic rings with an EC_50_ of 115 µM. In the uterus we found contractions to be relaxed with an EC_50_ of 1.3 µM. NaHS has been reported to relax different vascular tissues with EC_50_ of 1–300 µM [43], thus it is apparent that there is considerable inter-tissue differences in EC_50_ values, although experimental differences may account for much of the variation.

As mentioned earlier, studies in both animal and human tissues have demonstrated a role for H_2_S within smooth muscle. While many have reported relaxation [3], [4], [5], [12], some have found increased contraction or different effects dependent upon H_2_S concentration [4], [12]. It has been suggested that these differences may be due to the lower conversion efficacy of NaHS to H_2_S at high concentrations [26]. Such dual responses were not found by us with NaHS in pregnant rat myometrium, consistent with previous findings in the pregnant human myometrium [30]. *In vivo* data points to relaxation being the predominant effect of H_2_S in the vasculature. Mice lacking CSE, the biosynthetic enzyme for H_2_S, are hypertensive, their blood vessels do not relax to acetylcholine and administration of NaHS to animals causes vasodilation [44],. (but see also [45]).

### Effects of H_2_S Change with Gestational State

We found striking differences in the response of the myometrium, both in rat and human tissue, to GYY4137 and NaHS depending upon the gestational state of the tissue. We found no significant effects on contractions in the non-pregnant tissue to addition of either H_2_S source but clear effects by mid-gestation. The inhibitory effect on contraction then further increased until term. These data suggest that the relaxant effects of H_2_S are increased as pregnancy advances. The most striking effect however was the abrupt transition upon labour; H_2_S was without effect as seen by the data for both compounds. This suggests that H_2_S could contribute to uterine quiescence in later pregnancy and that the myometrium can rapidly change its responses to H_2_S.

### Mechanism of H_2_S Effects in Myometrium

There are many suggestions for the mechanism by which H_2_S exerts its effects and as with NO, it is likely that there will be many targets [8]. The main mechanism appears to be due to H_2_S modifying cysteine residues in many proteins through S-sulfhydration [46] i.e. cysteine’s covalent modification by which -SH groups on cysteine residues of a protein are converted to –S-SH., via addition of sulphur from H_2_S [47]. This molecular mechanism is similar to the S-nitrosylation effect of NO, however, unlike S-nitrosylation, S-sulfhydration activates rather represses, its target proteins [48]. The most widely researched effect of H_2_S is on K_ATP_ channels within smooth muscle. in vascular smooth muscle cells H_2_S stimulated single-channel activity of K_ATP_ channels by directly increasing their opening probability [41]. Recent work has made progress in identifying which residues in the channel are affected by H_2_S, with Cys 6 and 26 on the extracellular N terminal of the SUR1 subunit of the channel being identified [49]). K_ATP_ channels have been suggested as one of the targets of H_2_S that lead to reduced myometrial contractility [30]. In contrast, other studies showed that the K_ATP_ channels are not involved in H_2_S relaxation in smooth muscle tissues including vascular, bronchial, and gastrointestinal smooth muscle [3], [18], [50], [51]. Our data with glibenclamide would support a role for these channels in the mechanism of H_2_S effects in myometrium. Gyy4137 had no significant effect on myometrial contractility when K_ATP_ channels had been incubated with glibenclamide. As opening of these channels will cause hyperpolarization, and this in turn decreases the opening of L-type Ca channels, this suggests that K_ATP_ are a target in myometrium. Hyperpolarization and relaxation induced by Na_2_S has been directly measured in arterioles [52]. However as this hyperpolarization was shown to be due to activation of Ca sparks and opening of Ca-activated K (BK) channels, and Ca sparks are not present in myometrium [53], this cannot account for hyperpolarization in the myometrium.

### Changes in Intracellular Calcium

The above suggests that L-type Ca entry will be reduced by H_2_S sources in the uterus. There are however few studies measuring the effects of H_2_S on Ca in any tissues, and none have done so simultaneously with contraction. Reduction of Ca by H_2_S has previously been demonstrated in non-contractile arterial segments. [26]. Our simultaneous measurements of intracellular Ca and contractions show a H_2_S-dependent reduction in intracellular Ca accompanies the decrease in amplitude of the phasic contractions. To the best of our knowledge these are the first measurements directly demonstrating that the effects of H_2_S in producing reduction in force are due to decreased Ca transient amplitude.

### Effects of GYY4137 on Depolarized and Oxytocin-stimulated Contraction

We also show in pregnant rat myometrium that the tonic force produced by depolarization with high K, used to directly open L-type calcium channels, is reduced by GYY4137. This suggests that mechanisms beyond membrane potential changes are also a feature of the H_2_S relaxation mechanism in the uterus. There is mounting evidence that the L-type Ca channels themselves are targets of H_2_S. Sun et al, [54] in cardiac myocytes were the first to show that H_2_S can inhibit L-type Ca channels. Recently others have shown inhibition of these channels by NaHS also occurs in vascular smooth muscle [26], [55] and Zhang et al [25] have gone on to show that this is dependent upon the protein sulfhydryl state of the channel. An increase in resting Calcium was also found in endothelial cells with NaHS [56], thought to be due to store operated Ca entry. Thus direct effects on Ca entry via L-type Ca channels and other channels, also contributes to the relaxant effects of H_2_S. This inhibition of Ca channels will also explain why in some tissues inhibition with glibenclamide of K_ATP_ channels often does not fully prevent the relaxant effects of H_2_S donors. There is evidence from gastric fundus and distal colon, where glibenclamide is without effect, that H_2_S may affect Ca sensitization of the contractile machinery [5], [57], but as Ca sensitization plays little role in spontaneous activity of myometrium [58], this is unlikely to be contributing to the data we have obtained.

Our data clearly show that H_2_S donors can reduce contractility even when stimulated by oxytocin in the pregnant myometrium. Oxytocin increases Ca within the myometrium, partly by depolarization and increasing L-type Ca channel entry [59]. Thus the mechanisms by which H_2_S suppresses spontaneous contractions are likely to also feature in the effects on oxytocin-induced contractions. As shown in Table 2, the effects of NaHS were less in the presence of oxytocin compared to spontaneous activity, presumably due to the increased contractile drive with oxytocin stimulation.

### Gestational Changes and H_2_S Mechanism of Action

The above gives insight into the mechanism of action of H_2_S but does not explain the reasons for susceptibility changes over gestation. Suggestions include (i) increased vulnerability to sulfhydration of L-type calcium channels as L-type calcium channel subunits increase toward term [60]; (ii) reduction in kir6.1 and 6.2 K_ATP_ subunits once myometrium is labouring, as before labour H_2_S exerts its effects on these subunits [61]; (iii) up regulation of the H_2_S breakdown enzymes with gestation, or (iv) changes in uterine environment with labour, such as hypoxia an pH changes, [62], [63] may result in faster breakdown of H_2_S, but this remains controversial, [34], [64]. The lack of specific inhibitors of these enzymes and the difficulty of accurately measuring H_2_S in tissues hinders further study of these last two points.

### GYY4137 and Tocolysis

The synthesis of GYY4137 and its cardiovascular effects in rats were first reported by Li et al in 2008 [31]. As pointed out by these authors, while much data was being obtained showing the importance of H_2_S, studies were limited by the lack of a compound to better mimic the endogenous release of H_2_S in cells. The commonly used NaHS or Na_2_S release H_2_S instantaneously in aqueous solutions, producing very large and transient increases in its concentration. GYY4137’s potential as a slow-releasing H_2_S compound with effects on vascular smooth muscle *in vitro* and *in vivo* were shown, its time scale of H_2_S production measured and its lack of toxicity to aortic cells shown [31]. Subsequent work has supported low toxicity [65] and also indicated anti-cancer properties [42], anti inflammatory activity [66], and anti apoptotic [67] activity of GYY4137. Thus GYY4137 or subsequent compounds, [68] may be suitable for a variety of patho-physiological conditions, including tocolysis in threatened preterm labour, i.e. to stop the onset of labor, although further work, including studies on labouring samples, are needed to develop this suggestion. Recent studies of interactions between the enzymes producing or destroying H_2_S and their inhibitors, also represent another way of manipulating its effects in the uterus [69] The finding that free H_2_S values are up to 100-fold higher in smooth muscle (aorta) compared to liver, blood, heart and kidney, [70] also encourages these approaches.

### Summary

In conclusion, NaHS and GYY4137 relax term pregnant human and rat myometrium. Within the rat myometrium we show increased potency as term approaches; an effect that is rapidly reversed as labour starts. GYY4137 can reduce force produced spontaneously, by oxytocin or high K depolarization. The mechanism involves both K_ATP_ channels and importantly, L-type Ca channels. GYY4137 reduces the intracellular Ca transients that underlie spontaneous contractions. Our data and that of previous studies suggest H_2_S could contribute to uterine quiescence and that increasing its level in myometrium could be an attractive target for therapeutics to inhibit the onset of labor. Increased understanding of the mechanisms for transition to labor should also follow from obtaining a better understanding of H_2_S in the myometrium.
